# Shifts in waist-to-height ratio categories within tirzepatide groups: a post-hoc analysis of SURMOUNT-1

**DOI:** 10.1007/s40618-026-02883-7

**Published:** 2026-05-04

**Authors:** Naveed Sattar, Beverly G. Tchang, Royce P. Vincent, Hui Wang, Madhumita Murphy, Julia P. Dunn, Georgios K. Dimitriadis, Julia Fraseur Brumm

**Affiliations:** 1 School of Cardiovascular and Metabolic Health, University of Glasgow, Glasgow, UK; 2 Department of Medicine, Division of Endocrinology, Diabetes and Metabolism, Comprehensive Weight Control Center, Weill Cornell Medicine, New York, USA; 3 King’s College Hospital NHS Foundation Trust, London, UK; 4 Faculty of Life Sciences and Medicine, King’s College London, London, UK; 5 Eli Lilly and Company, Indianapolis, IN USA

**Keywords:** Body mass index, Cardiometabolic risk, Obesity, Waist circumference, Waist-to-height ratio, SURMOUNT-1, Tirzepatide

## Abstract

**Objective:**

To evaluate shifts in waist-to-height ratio (WHtR) categories among adults with obesity or overweight, with or without prediabetes, treated with tirzepatide in the SURMOUNT-1 study.

**Methods:**

This post hoc analysis included 2,538 participants from the SURMOUNT-1 Phase 3, double-blind, randomized, placebo-controlled trial. Adults with BMI ≥ 30 or ≥ 27 kg/m² and at least one obesity-related complication (ORC), excluding diabetes, were randomized to receive once-weekly tirzepatide (5, 10, or 15 mg) or placebo, alongside a reduced-calorie diet and increased physical activity. Participants were grouped by baseline WHtR (≤ 0.49, > 0.49 to ≤ 0.59, > 0.59) according to the National Institute for Health and Care Excellence (NICE) framework. Participants with prediabetes at baseline had additional follow-up data beyond week 72, and shifts in their WHtR categories at week 176 were also included. Change from baseline in WHtR was analyzed using a mixed model for repeated measures (MMRM). Shift tables were used to summarize changes from baseline to post-baseline WHtR category levels.

**Results:**

At baseline, 89.8% of participants had a WHtR > 0.59, 10.1% had > 0.49 to ≤ 0.59, and 0.1% had ≤ 0.49. After 72 weeks of tirzepatide (10/15 mg), 16.7% of participants achieved a WHtR ≤ 0.49, and 54.7% improved their baseline WHtR category compared to 9.6% with placebo. At 176 weeks, among participants with prediabetes, 12.2% achieved WHtR ≤ 0.49, and 46.4% improved their category with tirzepatide versus 9.3% with placebo.

**Conclusions:**

Tirzepatide treatment was associated with sustained improvements in WHtR categories, with a greater proportion of participants shifting to a better WHtR category compared to participants treated with placebo. Improved WHtR may be suggestive of lower future cardiometabolic risk. Further analyses of this nature will enhance the understanding and application of WHtR in obesity management across diverse populations.

**Supplementary Information:**

The online version contains supplementary material available at 10.1007/s40618-026-02883-7.

## Introduction

Obesity, a well-established contributor to morbidity and mortality, is a global epidemic that requires scalable approaches for disease diagnosis, treatment, and monitoring [1, 2]. For decades, obesity management clinical guidelines have recommended assessing health risk using anthropometric measures such as body mass index (BMI) and waist circumference.

BMI has long been used as a practical and scalable surrogate to predict excess adiposity; however, BMI does not necessarily indicate the impact of excess adiposity on an individual, and BMI’s association with health risk, perhaps particularly when BMI is in the overweight category, can be inconsistent and varies with age, sex, and ethnicity [3–6]. Additionally, the utility of BMI alone to assess health risk is limited in the context of weight reduction interventions and among individuals where central adiposity predominates [7]. Treatment algorithms based on practical anthropometric measures, such as BMI and waist circumference, are used to guide risk prediction and intervention strategies to recommend medications for obesity management [8]. For individuals with a BMI of 25.0–29.9 kg/m², lifestyle interventions are recommended. Additionally, pharmacotherapy may be considered if co-morbidities are present and treatment goals are not achieved with lifestyle modifications alone [8–10]. For BMI 30.0–34.9 kg/m², drug therapy may be added, especially if presence of abdominal obesity, or if obesity related complications are present. At BMI 35.0–39.9 kg/m², surgery is considered alongside drug therapy and lifestyle changes if comorbidities are present, and for BMI > 40.0 kg/m², all three approaches – lifestyle, drug therapy, and surgery are advised [11]. However, using measures that combine BMI and waist circumference can be limited by variability across populations and a lack of standardization, which ultimately restricts their application in clinical settings [12–14].

The National Institute for Health and Care Excellence (NICE) has recommended using BMI as a measure of overweight and obesity but has also recommended that results be interpreted with caution. For adults with a BMI < 35 kg/m^2^, NICE guidelines recommend using the waist circumference to height ratio (WHtR) in addition to BMI as a practical estimate of central adiposity, and with better discriminatory and predictive power for cardiometabolic disease outcomes such as type 2 diabetes than BMI alone [15]. Since changes in WHtR have not been shown to predict lower risk independent of change in BMI, the combination of BMI and WHtR may be used to assess and predict health risks [16].

Similarly, the European Association for the Study of Obesity (EASO) has published a framework for diagnosing, staging, and managing obesity that more accurately reflects obesity as an adiposity-based chronic disease [10]. The EASO framework uses anthropometric components such as BMI and WHtR alongside clinical components of obesity (medical, functional, and mental domains) to diagnose and manage obesity. This framework includes individuals with lower BMI (≥ 25–30 kg/m^2^) and increased abdominal fat accumulation and ORCs within the definition of obesity [10]. Additionally, it is recommended that waist circumference be measured in those with a BMI below 35 kg/m^2^ to help identify visceral adiposity and assess increased cardiometabolic disease risk [17, 18].

Given the recent importance placed on anthropometric measures of obesity beyond BMI, WHtR has emerged as a potential, evidence-based tool for diagnosing and treating obesity, as well as serving as a tool to better assess a multitude of health risks [16]. The recommendations for the utility of WHtR are supported by many years of evidence, including studies in diverse populations, and its use is being considered in clinical practice, not only for diagnosing obesity but also for assessing comorbid health risks [14, 16, 19–22]. In fact, the need for additional measurements is subverted by deriving WHtR from waist circumference and BMI data. Since WHtR is non-invasive and affordable, it can be a key tool in the early identification and intervention for those with obesity or overweight and ORCs [23]. However, little data exist on changes in WHtR with newer approved medications for weight loss.

The SURMOUNT-1 study evaluated the efficacy and safety of tirzepatide in adults with obesity or overweight who did not have diabetes and showed that tirzepatide led to substantial and sustained reductions in body weight [24]. The current post hoc analysis of SURMOUNT-1 evaluated WHtR shifts from baseline, at the primary 72-week endpoint, and in the prediabetes subgroup at the 176-week endpoint.

## Methods

### Study design

The SURMOUNT-1 study design has been previously published [24, 25]. Briefly, SURMOUNT-1 was a Phase 3, double-blind, randomized, controlled trial that included adults with a BMI ≥ 30 or ≥ 27 and at least one ORC, excluding diabetes. Participants were randomly assigned 1:1:1:1 to receive once-weekly subcutaneous tirzepatide (5 mg, 10 mg, 15 mg) or placebo, alongside lifestyle intervention. Treatment duration was determined by prediabetes status at baseline; those with obesity and without prediabetes at baseline were enrolled in the study for 72 weeks, while those with obesity and prediabetes at baseline continued in the extension study involving a total of 176 weeks of treatment [24]. Prediabetes was defined by the presence of at least two abnormal values among glycated hemoglobin (HbA1c), fasting glucose, and 2-hour oral glucose tolerance test (OGTT) [25].

### Key measures and analyses

This post hoc analysis included 2538 participants who had a baseline WHtR measurement, of whom 1,032 had prediabetes at baseline. Participants randomized to tirzepatide 10 mg and 15 mg were pooled (tirzepatide 10/15 mg) for the purposes of this analysis. Waist circumference was measured in the horizontal plane at the midpoint between the lower margin of the last palpable rib and the top of the iliac crest. WHtR was calculated as waist circumference (in cm) divided by height (in cm), and participants were grouped based on their WHtR at baseline: ≤0.49, > 0.49 to ≤ 0.59, and > 0.59, with values exactly at the threshold classified into the lower (healthier) category. These categories are aligned with the NICE framework, which defines healthy central adiposity as a WHtR of 0.4 to 0.49, increased central adiposity as 0.5 to 0.59, and high central adiposity as 0.6 or more [15]. The shifts from these baseline WHtR categories to a participant’s category at Week 72 and Week 176 were analysed using the efficacy analysis set (the intention-to-treat population).

Change from baseline in WHtR was analyzed using a mixed model for repeated measures (MMRM), with change from baseline at each scheduled post-baseline visit during the treatment period as the dependent variable. The analysis population included all subjects with a non-missing baseline value and at least one non-missing post-baseline assessment of WHtR. The model included fixed effects for baseline WHtR, treatment, visit, treatment-by-visit interaction, country, and sex. Prediabetes status at randomization was also included as a covariate to the model applied to overall population for the Week 72 endpoint. Visit was treated as a categorical variable. An unstructured covariance matrix was used to model within-subject errors. Shift tables were used to summarize changes from baseline to post-baseline WHtR category levels. Baseline category was defined using the last non-missing assessment prior to the first dose of study treatment. Post-baseline category was defined as the last non-missing assessment recorded during the treatment period. Participants without a post-baseline WHtR assessment were retained in the analysis and categorized as “missing” in the shift tables. Shift tables present the cross-classification of baseline and post-baseline categories. Analyses were descriptive in nature, and no formal hypothesis testing was performed for category-shift endpoints.

## Results

At Week 72, the least squares mean (SE) change from baseline in WHtR was − 0.09 (0.003) in participants treated with 5 mg tirzepatide and − 0.12 (0.002) in participants treated with 10 or 15 mg tirzepatide, compared to −0.02 (0.003) in the placebo group (Online Resource: Table S1). At Week 176, which included only those participants with prediabetes at baseline, the change from baseline in WHtR was − 0.08 (0.005) and − 0.12 (0.003) in participants treated with 5 mg tirzepatide and 10 or 15 mg tirzepatide, respectively, compared to −0.01 (0.005) in the placebo group at Week 176.

At baseline, 2 participants (0.1%) had a WHtR ≤ 0.49, while 256 participants (10.1%) had a WHtR > 0.49 but ≤ 0.59, and 2280 participants (89.8%) had a baseline WHtR > 0.59. The majority of participants were classified as being at high risk at baseline based on WHtR. After 72 weeks of treatment with 10 or 15 mg tirzepatide, 212 participants (16.7%) had a WHtR ≤ 0.49, and at 176 weeks, 63 participants (12.2%) had a WHtR ≤ 0.49 (Table 1).

**Table 1:**
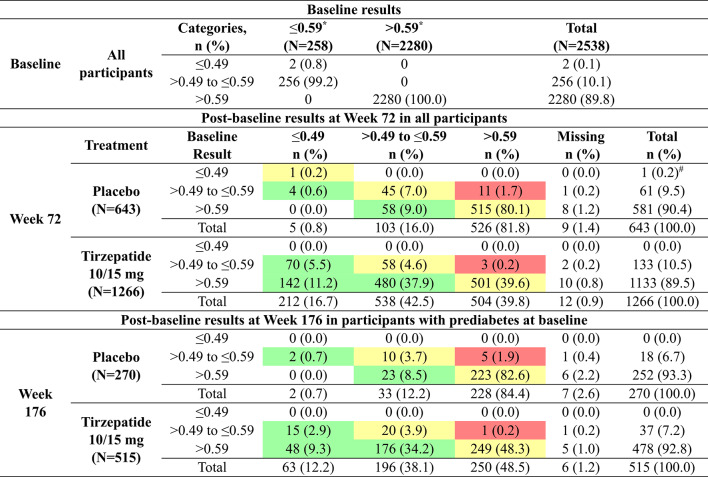
WHtR shift tables from baseline to Week 72 and Week 176

Number and percentage of participants at each WHtR category and baseline, Week 72, and Week 176. Participants who improved WHtR categories are highlighted in green, those who maintained WHtR categories are in yellow, and those who worsened are in red

The postbaseline category was defined as the last non-missing WHtR assessment recorded during the treatment period at the respective time point (Week 72 or Week 176)

*WHtR was grouped into three categories: ≤0.49, > 0.49 and ≤0.59, and >0.59. Due to the small numbers of ≤0.49 category at baseline, the combined category of ≤0.59 and >0.59 is reported at baseline

#One participant with a WHtR of ≤0.49 was part of the tirzepatide 5 mg treatment group

n, number of participants in the analysis (subset) population; N, total number of participants; WHtR, waist-to-height ratio

**Fig. 1:**
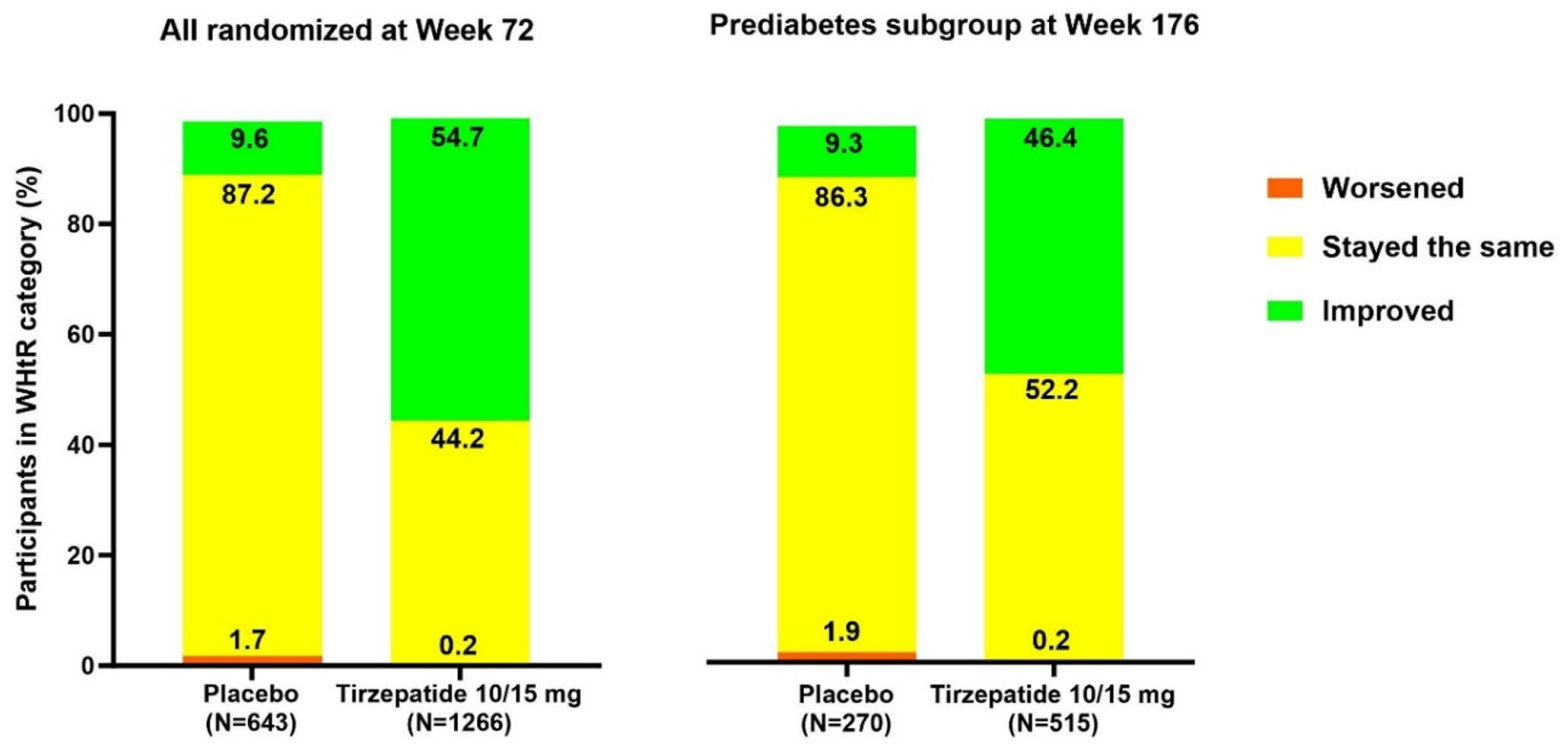
WHtR shifts from baseline in participants treated with 10 or 15 mg tirzepatide. Week 72 shows post-baseline results in all participants. Week 176 shows post-baseline results in participants with prediabetes at baseline. Percentage of participants who shifted or maintained WHtR categories at Week 72 and Week 176 as compared with baseline. Categories may not sum to 100% due to missing data. n, number of participants in the analysis (subset) population; N, total number of participants;WHtR, waist-to-height ratio

Following treatment with tirzepatide, over half of the participants improved WHtR categories, as seen in Fig. 1. At Week 72, 54.7% of participants treated with 10 mg or 15 mg tirzepatide improved WHtR categories compared with 9.6% of participants treated with placebo, and 17% achieved a normal WHtR compared to just 0.6% with placebo. At Week 176, among participants with prediabetes at baseline, 46.4% of those treated with 10 or 15 mg of tirzepatide improved WHtR categories compared to 9.3% of participants treated with placebo. Additionally, fewer participants treated with 10 mg or 15 mg worsened (W72: 0.2%, W176: 0.2%) compared with those treated with placebo (W72: 1.7%, W176: 1.9%).

Tirzepatide 5 mg also showed improvement over placebo, with 44.9% of participants improving WHtR categories at Week 72 compared to 9.6% with placebo. Among participants with prediabetes, 38.9% achieved at least one categorical improvement at Week 176 versus 9.3% with placebo (Online resource: Figure S1).

## Conclusions

Treatment with tirzepatide was associated with over half of the participants achieving an improvement in WHtR classification compared to less than 10% with placebo at week 72. This benefit was sustained at week 176 in participants with prediabetes who received treatment with tirzepatide, as 46.4% of participants experienced an improvement in WHtR classification, compared to 9.3% with placebo. In this post hoc analysis, tirzepatide treatment was associated with meaningful improvements in WHtR categories relative to placebo. Incorporating WHtR into the clinical diagnosis of excess adiposity and the treatment of obesity may provide a practical tool that better draws attention to future health improvements, though long-term cardiometabolic outcome data would be needed to best direct treatment goals. The observed changes in WHtR are consistent with previous evidence demonstrating clinically meaningful and sustained weight reduction with tirzepatide [24]. However, the long-term WHtR findings at Week 176 were derived exclusively from participants with prediabetes at baseline and should not be extrapolated to the full SURMOUNT-1 cohort, of which about 60% did not have prediabetes. Future investigations should consider reporting rates of BMI and WHtR improvements according to ethnic subgroups to provide additional information on the relative utility of each anthropometric measure. Additional studies are important to elucidate the health impact of WHtR, particularly in relation to changes induced by potent therapeutic interventions.

## Supplementary Information

Below is the link to the electronic supplementary material.

Supplementary Material 1

## Data Availability

Lilly provides access to all individual participant data collected during the trial, after anonymization, with the exception of pharmacokinetic or genetic data. Data are available to request 6 months after the indication studied has been approved in the US and EU and after primary publication acceptance, whichever is later. No expiration date of data requests is currently set once data are made available. Access is provided after a proposal has been approved by an independent review committee identified for this purpose and after receipt of a signed data sharing agreement. Data and documents, including the study protocol, statistical analysis plan, clinical study report, blank or annotated case report forms, will be provided in a secure data-sharing environment. For details on submitting a request, see the instructions provided at www.vivli.org.
