# Supplementary material for: Shifts in waist-to-height ratio categories within tirzepatide groups: a post-hoc analysis of SURMOUNT-1
Source: J Endocrinol Invest. 2026 May 4;49(6):1237–43. doi: 10.1007/s40618-026-02883-7 (PMC13219146; doi:10.1007/s40618-026-02883-7)
Supplement: Supplementary file 1 — Supplementary Material 1 [file 40618_2026_2883_MOESM1_ESM.docx]

# **Title page**

**Title: Shifts in waist-to-height ratio categories within tirzepatide groups: A post-hoc analysis of SURMOUNT-1**

**Running title:** Waist-to-height ratio shifts in SURMOUNT-1

**Authors list**

Naveed Sattar^1^, Beverly G. Tchang^2^, Royce P. Vincent^3,4^, Hui Wang^5*^, Madhumita Murphy^5^, Julia P. Dunn^5^, Georgios K. Dimitriadis^5^, Julia Fraseur Brumm^5^

**Affiliations**

^1^School of Cardiovascular and Metabolic Health, University of Glasgow, Glasgow, UK

^2^Department of Medicine, Division of Endocrinology, Diabetes and Metabolism, Comprehensive Weight Control Center, Weill Cornell Medicine, New York, USA

^3^King's College Hospital NHS Foundation Trust, London, UK

^4^Faculty of Life Sciences and Medicine, King's College London, UK

^5^Eli Lilly and Company

^*^The author was an employee of Eli Lilly and Company during the study period.

**Author details:**

1. Naveed Sattar

School of Cardiovascular and Metabolic Health, University of Glasgow, Glasgow, UK

Email address: [Naveed.Sattar@glasgow.ac.uk](mailto:Naveed.Sattar@glasgow.ac.uk)

1. Beverly G. Tchang

Department of Medicine, Division of Endocrinology, Diabetes and Metabolism, Comprehensive Weight Control Center, Weill Cornell Medicine, New York, USA

Email address: [bgt9001@med.cornell.edu](mailto:bgt9001@med.cornell.edu)

1. Royce P. Vincent

King's College Hospital NHS Foundation Trust, London, UK, Faculty of Life Sciences and Medicine, King's College London, UK

Email address: [royce.vincent@nhs.net](mailto:royce.vincent@nhs.net)

1. Hui Wang

Eli Lilly and Company, Indianapolis, IN, USA

Email address: [hwang@fcrinstitute.org](mailto:hwang@fcrinstitute.org)

1. Madhumita Murphy

Eli Lilly and Company, Indianapolis, IN, USA

Email address: [murphy_madhumita@lilly.com](mailto:murphy_madhumita@lilly.com)

1. Julia P. Dunn

Eli Lilly and Company, Indianapolis, IN, USA

Email address: dunn_julia@lilly.com

1. Georgios K. Dimitriadis

Eli Lilly and Company, Indianapolis, IN, USA

Email address: [georgios.dimitriadis@lilly.com](mailto:georgios.dimitriadis@lilly.com)

1. Julia Fraseur Brumm

Eli Lilly and Company, Indianapolis, IN, USA

Email address: [fraseur_julia@lilly.com](mailto:fraseur_julia@lilly.com)

***Corresponding author**

Julia Fraseur Brumm,

Eli Lilly and Company, Indianapolis, IN, USA

Email: [fraseur_julia@lilly.com](mailto:fraseur_julia@lilly.com)

**Supplements:** Table – 1; Figure – 1

**Table S1. Mean change in WHtR in participants treated with 5 mg tirzepatide or 10 or 15 mg tirzepatide**

| **Parameter** | **Placebo** | **Tirzepatide 5 mg** | **Tirzepatide 10 or 15 mg** |
| --- | --- | --- | --- |
| **WHtR in all participants** | | | |
| **WHtR at baseline, n** | n=634 | n=622 | n=1254 |
| Mean (SD) | 0.69 (0.09) | 0.68 (0.08) | 0.69 (0.09) |
| **WHtR at Week 72, n** | n=471 | n=538 | n=1065 |
| Mean (SD) | 0.66 (0.09) | 0.59 (0.09) | 0.57 (0.09) |
| **Change in WHtR at Week 72** |  |  |  |
| LSM change from baseline (SE) | -0.02 (0.003); p<0.001 | -0.09 (0.003); p<0.001 | -0.12 (0.002); p<0.001 |
| LSM difference from placebo (95%CI) | - | -0.07 (-0.08 to -0.06); p<0.001 | -0.10 (-0.11 to -0.09); p<0.001 |
| **WHtR in participants with prediabetes at baseline** | | | |
| **WHtR at baseline, n** | n=263 | n=244 | n=509 |
| Mean (SD) | 0.71 (0.09) | 0.69 (0.08) | 0.70 (0.09) |
| **WHtR at Week 176, n** | n=114 | n=142 | n=315 |
| Mean (SD) | 0.68 (0.09) | 0.61 (0.09) | 0.59 (0.10) |
| **Change in WHtR at Week 176** |  |  |  |
| LSM change from baseline (SE) | -0.01 (0.005); p=0.005 | -0.08 (0.005); p<0.0001 | -0.12 (0.003); p<0.0001 |
| LSM difference from placebo (95%CI) | - | -0.06 (-0.08 to -0.05); p<0.0001 | -0.10 (-0.11 to -0.09); p<0.0001 |

Only subjects with a non-missing baseline value and at least one non-missing post-baseline value of the response variable were included in the analysis.

LSMs were from the MMRM model: change in WHtR = baseline value + country + sex + treatment + time + treatment*time + prediabetes status at randomization for the Week 72 endpoint. Variance-Covariance structure = Unstructured.

Abbreviations: CI, confidence interval; LSM, least squares mean; n, number of participants in the analysis (subset) population; SD, standard deviation; SE, standard error; WHtR, waist-to-height ratio.

**Fig. S1 WHtR shifts from baseline in participants treated with 5 mg tirzepatide**


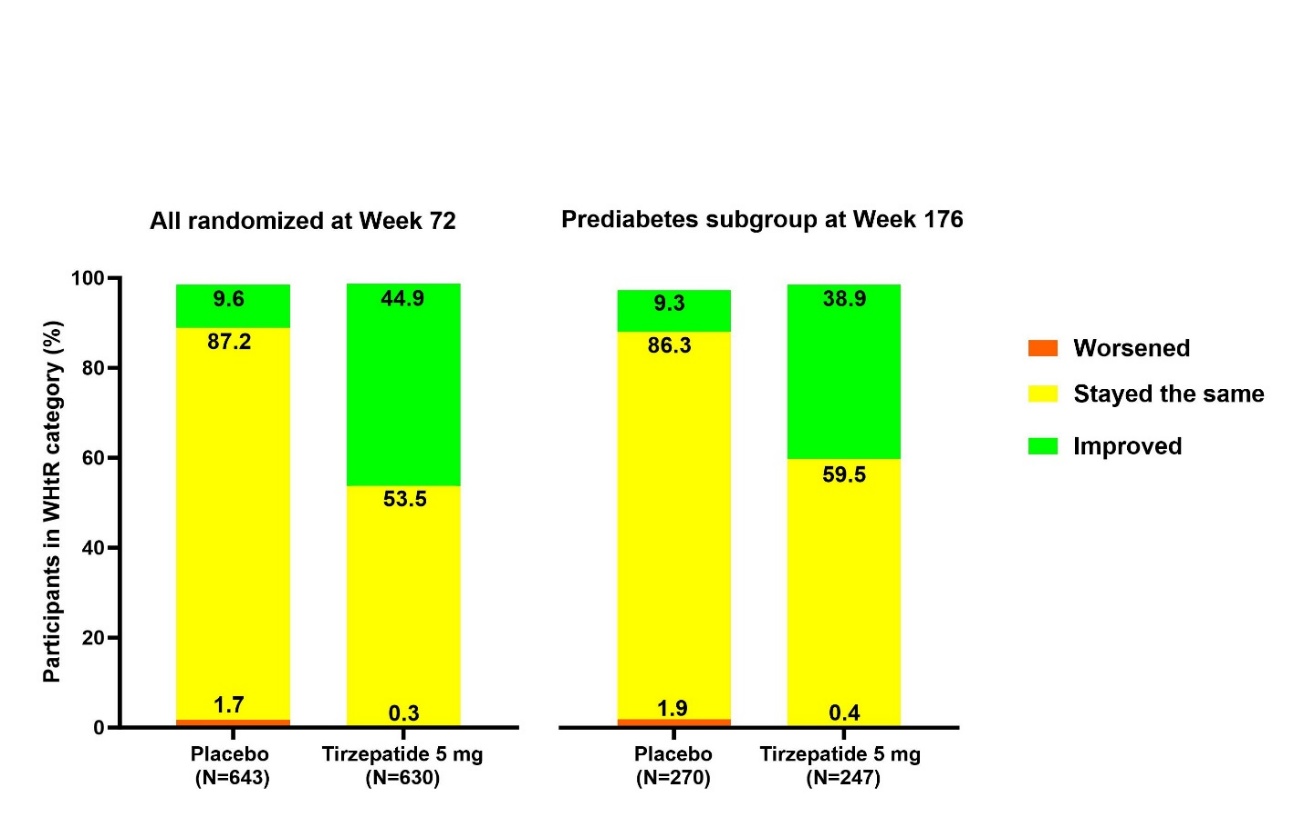
Week 72 shows post-baseline results in all participants. Week 176 shows post-baseline results in participants with prediabetes at baseline. Percentage of participants who shifted or maintained WHtR categories at Week 72 and Week 176 as compared with baseline. Categories may not sum to 100% due to missing data.

Abbreviations: n, number of participants in the analysis (subset) population; N, total number of participants; WHtR, waist-to-height ratio.
